# STAT3-Inducible Mouse ESCs: A Model to Study the Role of STAT3 in ESC Maintenance and Lineage Differentiation

**DOI:** 10.1155/2018/8632950

**Published:** 2018-09-04

**Authors:** Yu Qian Wong, Hongyan Xu, Qiang Wu, Xinyu Liu, Chengchen Lufei, Xiu Qin Xu, Xin-Yuan Fu

**Affiliations:** ^1^Department of Biochemistry, Yong Loo Lin School of Medicine, National University of Singapore, 8 Medical Drive, Singapore 117597; ^2^Cancer Science Institute of Singapore, National University of Singapore, 14 Medical Drive, Singapore 117599; ^3^Key Laboratory of Tropical & Subtropical Fishery Resource Application & Cultivation of Ministry of Agriculture, Pearl River Fisheries Research Institute, Chinese Academy of Fishery Sciences, Guangzhou 510380, China; ^4^The State Key Laboratory of Quality Research in Chinese Medicine, Macau University of Science and Technology, Room 704a, Block H, Avenida Wai Long, Taipa, Macau; ^5^Institute of Stem Cell and Regenerative Medicine, Medical College, Xiamen University, Xiamen, Fujian 361100, China; ^6^Department of Biology, Southern University of Science and Technology, 1088 Xueyuan Ave, Nanshan Qu, Shenzhen Shi, Guangdong Sheng 518055, China

## Abstract

Studies have demonstrated that STAT3 is essential in maintaining self-renewal of embryonic stem cells (ESCs) and modulates ESC differentiation. However, there is still lack of direct evidence on STAT3 functions in ESCs and embryogenesis because constitutive STAT3 knockout (KO) mouse is embryonic lethal at E6.5–E7.5, prior to potential functional role in early development can be assessed. Therefore, in this study, two inducible STAT3 ESC lines were established, including the STAT3 knockout (InSTAT3 KO) and pSTAT3 overexpressed (InSTAT3 CA) using Tet-on inducible system in which STAT3 expression can be strictly controlled by doxycycline (Dox) stimulation. Through genotyping, deletion of STAT3 alleles was detected in InSTAT3 KO ESCs following 24 hours Dox stimulation. Western blot also showed that pSTAT3 and STAT3 protein levels were significantly reduced in InSTAT3 KO ESCs while dominantly elevated in InSTAT3 CA ECSs upon Dox stimulation. Likewise, it was found that STAT3-null ESCs would affect the differentiation of ESCs into mesoderm and cardiac lineage. Taken together, the findings of this study indicated that InSTAT3 KO and InSTAT3 CA ESCs could provide a new tool to clarify the direct targets of STAT3 and its role in ESC maintenance, which will facilitate the elaboration of the mechanisms whereby STAT3 maintains ESC pluripotency and regulates ESC differentiation during mammalian embryogenesis.

## 1. Introduction

Signal transducer and activator of transcription 3 (STAT3) belongs to the STAT family and is the only member essential for early embryo development since STAT3 null mice were found to be embryonic lethal at E6.5 to E7.5 [1]. Subsequently, tissue-specific functions of STAT3 have been well studied using Cre-loxP recombination system driven by discrete promoter in specific tissues to address the pivotal role of STAT3 in mammalian organogenesis. Tissue-specific gene targeting demonstrated that STAT3 functions in various physiological activities, including wound healing in keratinocytes, mammary involution, regeneration of liver, survival of neuron cells, development of Th17 and T cell proliferation, and cytoprotection of respiratory epithelium during adenoviral infection [2–8]. Moreover, it has been demonstrated that STAT3 is a well-known transcription factor participating in a wide variety of biological processes, such as embryonic stem cell pluripotency maintenance, embryogenesis, cardioprotection, cancer, and immunity [1, 9–12].

STAT3 is an essential mediator downstream of leukemia inhibitory factor (LIF) in maintaining ESC pluripotency. Studies have proved that STAT3 activation is essential to maintain ESC self-renewal [13–15]. In the absence of LIF, constitutive activation of STAT3 is sufficient to inhibit mouse ESC differentiation [15]. Through a conditional active STAT3 system, it was found that STAT3 activation is adequate to maintain the self-renewal of ESCs [15]. However, it is unclear how downstream targets of LIF/JAK/STAT3 signaling play a preeminent role in maintaining ESC pluripotency by interacting with the master regulators of pluripotency factors for ESCs, namely, Oct4, Nanog, and Sox2 [16, 17]. Until recently, STAT3 has been reported that it directly regulates Oct4 and Nanog transcription to maintain pluripotency of ESCs and iPSCs. Knockdown of STAT3 in ESCs leads to substantial reduction of Oct4 expression. Furthermore, STAT3 binds directly to the distal enhancer of Oct4 and Nanog and positively regulates Oct4 and Nanog to maintain pluripotency of ESCs [18]. In contrast, it has previously been observed that interruption of STAT3 leads to ESC differentiation [13, 19]. Expression of STAT3 dominant-negative mutant using an inducible promoter in ESCs abolishes the self-renewal of ESCs as maintained by LIF and promotes differentiation [13, 14].

In addition to maintenance of ESC self-renewal and pluripotency, STAT3 also implemented a role in embryogenesis and cell-fate determination. STAT3 is highly expressed in mouse oocytes and becomes phosphorylated and translocates into the nucleus in the four-cell and later stage embryos, indicating that activated STAT3 is present in preimplantation embryos [18]. During mouse embryogenesis, high-level expression of STAT3 mRNA initiates at around E7.5 in the embryo itself. Subsequently, STAT3 mRNA was identified in a number of tissues including the yolk sac endoderm, myometrium, cephalic mesenchyme, and blood islands by E9.5 [20]. These findings suggest that STAT3 is crucial to early embryogenesis, as well as the subsequent differentiation into various cell lineages including the development of many specific organs such as liver development, myeloid cell differentiation, skin remodeling, angiogenesis, and astrogenesis requires STAT3 activation [2, 21–24].

STAT3 conventional all-tissue knockout mice have previously been found early embryonic lethal at E6.5 to E7.5, as a result of embryos rapidly degenerated between E6.5 and E7.5 with no obvious mesoderm formation [1]. In fact, STAT3 ablation resulted in embryonically lethal less than 1 day before the embryo would develop into beating cardiomyocytes at E7.5 to E8.5 [1, 25–27]. Earliest evidence revealed that STAT3 was confined to areas within the embryo, including the presumptive heart field, and was activated when cardiomyocytes would be occurring at E7.5 to E8.5 [20]. Thus, in recent years, increasing studies have documented the importance of STAT3 in initiation of cardiomyogenesis. Yet, few studies have explored the role of STAT3 in early cardiomyocyte differentiation because conventional STAT3 KO is embryonic lethal at the time of formation of cardiomyocyte at E7.5–E8.5 and before a potential functional role in cardiac differentiation can be assessed. Therefore, the mechanisms by which STAT3 regulates early cardiomyocyte differentiation have not yet been well established.

ESCs originate from the inner cell mass of blastocyst, which is an early stage of preimplantation embryo [28, 29]. ESCs are pluripotent cells with a capacity to differentiate to all cell types of the adult body and they can be cultured indefinitely *in vitro* through self-renewing division [30–32]. Therefore, ESCs are suitable for studying the initiation of mammalian organogenesis through differentiating ESCs into defined cell types [33–37].

Hence, in this study, an *in vitro* inducible STAT3 KO and STAT3 CA system of mouse ESCs was constructed where STAT3 expression can be strictly controlled by Dox adding or withdrawing. The present study attempts to utilize InSTAT3 KO ESCs to demonstrate the role of STAT3 in cardiac lineage differentiation and in the initiation of cardiac differentiation from ESCs. The findings will provide a new tool to elaborate the functions of STAT3 in ESC pluripotency and differentiation into certain lineages.

## 2. Materials and Methods

### 2.1. ESC Culture

ESCs were maintained in undifferentiated state with Glasgow Minimum Essential Medium (Gibco, Grand Island, New York, USA), supplemented with 15% ES-qualified fetal bovine serum (Gibco, USA), 1 mM sodium pyruvate (Gibco, Grand Island, New York, USA), 0.1 mM nonessential amino acids (Gibco, Grand Island, New York, USA), 0.1 mM 2-mercaptoethanol (Sigma-Aldrich, Saint Louis, Missouri, USA), 50 U/ml penicillin (Gibco, Grand Island, New York, USA), 50 ug/ml streptomycin (Gibco, Grand Island, New York, USA), and 1000 U/ml murine LIF (ESGRO, Millipore, Chemicon, USA) on gelatin-coated culture dish at 37°C in humidified air with 5% CO_2_. Medium was changed every two days and the cells were subcultured when they reached 70–80% confluency. Cells were made to undergo two passages after thawing before commencement of experiments.

### 2.2. Generation of Inducible STAT3 KO and pSTAT3 overexpressed ESCs

The 3rd generation Tet-on System was purchased from Clontech Laboratories (Cat. number 631167) (A Takara Bio Company, CA, USA). This Tet-On 3G System is an inducible gene expression system for mammalian cells. The system consists of the Tet-On 3G transactivator, TetR (Figure S1A), and contains a gene of interest (GOI) under the control of a TRE3G promoter (pTRE3G) (Figure S1B, 1C) which will express high levels of GOI driven by TetR with Dox induction [38–41].

Full length of mouse wild-type STAT3 cDNA was cloned from ESCs cDNA pool by PCR, constitutive activated STAT3 (CA-STAT3) or overexpression of pSTAT3 was obtained from pXJ40-CA (kindly provided by Professor Cao Xinmin) [42], and NlsCre was purchased from commercial entities. CA-mSTAT3 and NlsCre were cloned into a TetR response vector controlled by the tetracycline responsive element (pTRE-), fused with C-T2ACherry and C-IRESzsGreen, respectively (Figure S1).

### 2.3. Transfection

Transfection was done when cells were grown to 70–80% of confluency. Cells were transfected by Lipofectamine 2000 (Life Technologies, Carlsbad, CA, USA), and the manufacturer's protocol was followed. 5 *μ*g of plasmid DNA was diluted in Opti-MEM medium (Gibco, Grand Island, New York, USA) for a final volume of 50 *μ*l. 2 *μ*l of Lipofectamine 2000 was diluted in 48 *μ*l Opti-MEM medium followed by a 5 min incubation at room temperature. Diluted DNA was added to Lipofectamine 2000 diluted at 1 : 1 ratio and incubated for 20 min at room temperature. After incubation, the DNA/Lipofectamine mixture was added to each well and gently swirled to mix. The transfected cells were incubated at 37°C with 5% CO_2_. After about 4 to 6 hours, media was removed and replaced with fresh media. Then, cells were moved back to 37°C incubator with 5% CO_2_ until cells reached full confluency for harvest, and the transfection efficiency was checked by viewing in a fluorescent microscopy.

### 2.4. Genotyping

The STAT3 deleted (STAT3^D^) PCR amplification protocol consisted of one initial denaturation step for 5 minutes at 95°C, followed by 35 cycles: denaturation (95°C for 30 s), annealing for 30 s at 65°C, and extension at 72°C for two minutes. The PCR amplified samples were subjected to subsequent gel electrophoresis. 2% agarose gel was used to visualize the STAT3^D^ band. The gel electrophoresis was run at the voltage 100 volt for about 40 minutes. The gel was then viewed under UV. The size of gene segment was determined by 100 base pair DNA ladder (Thermo Fisher Scientific, Carlsbad, CA, USA). List of primers used in genotyping are included in Table S1.

### 2.5. RNA Isolation and Quantitative Real-Time PCR (qRT-PCR)

Total RNA was extracted using TRIzol reagent (Thermo Fisher Scientific, Carlsbad, CA, USA) according to the manufacturer's instructions, purified by the RNeasy mini kit (Qiagen, Hilden, Germany), and then reverse transcribed using the M-MLV Reverse Transcriptase system (Promega, Madison, WI, USA) into cDNAs. The cDNA products were used for semiquantitative RT-PCR with KAPA SYBR® FAST Universal 2X qPCR Master Mix (KK4600) (Kapa Biosystems, Cape Town, South Africa) using a 7300 Real-Time PCR machine (Applied Biosystems). All gene-expression analyses were normalized against the housekeeping gene GAPDH. The sequences of primers used are included in Table S2. Triplicate was done for the analysis of each samples.

### 2.6. Protein Extraction and Western Blotting

Cells were washed twice with ice-cold PBS and lysed in whole cell extract buffer supplemented with protease inhibitor (Roche, Indianapolis, USA). The supernatant was collected by centrifugation, and protein concentrations were measured with BCA assay (Bio-Rad, CA, USA). The absorbance of the proteins in the lysates was then measured using GeneQuant 1300 machine with wavelength set at 595 nm. Appropriate amount of lysate samples was mixed with sample buffer, separated by SDS-polyacrylamide gel electrophoresis of a gel percentage dependent on the size of the target protein, and transferred onto a polyvinylidene difluoride (PVDF) membrane (Merck Millipore, Darmstadt, Germany) in cold room. After blocking with 5% nonfat milk in washing buffer, the membranes were incubated with the indicated primary antibodies (Table S3) at 4°C overnight. Following washes, they were incubated in horseradish peroxidase- (HRP-) conjugated secondary antibodies. The immunoreactive bands were visualized using SuperSignal West Pico Chemiluminescent Substrate on audiographic film (Thermo Scientific, Rockford, USA) that was developed by a film cassette. Equal loading the blots was shown by *β*-actin level.

### 2.7. Immunofluorescent Staining

Slides were washed with PBS solution, fixed with 4% paraformaldehyde (Sigma-Aldrich, Saint Louis, Missouri, USA) for 10 min at room temperature, and permeabilized with 0.2% Triton X-100 (Sigma-Aldrich, Saint Louis, Missouri, USA) in PBS for 10 min. Then slides were blocked with 3% BSA (Sigma-Aldrich, Saint Louis, Missouri, USA) in PBS with 0.1% Triton X-100 for 10 min, followed by incubation with indicated primary antibodies (Table S3) at 4°C overnight. Then slides were washed three times with 0.1% Triton X-100 in PBS, each time for 10 min and incubated with fluorescence-labeled secondary antibodies in dark at room temperature for 1 hour. Nuclei were visualized by DAPI (Molecular Probes, Eugene, Oregon, USA) staining. Coverslips were mounted onto slides by VECTASHIELD HardSet Mounting Medium (Vector Laboratories Inc., Burlingame, CA, USA). Labeled sections were imaged using Nikon A1R-A1 confocal microscopy system.

### 2.8. ESC Cardiac Differentiation

**Figure 1:**
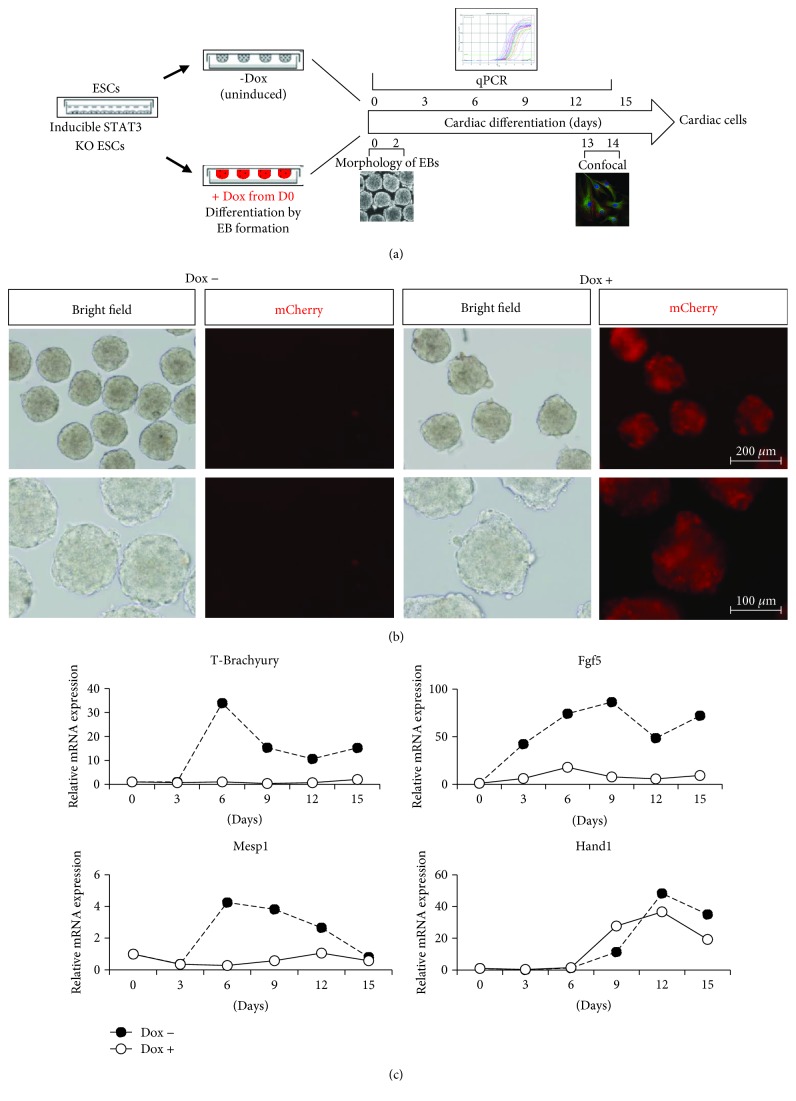
Use of InSTAT3 KO ESCs to study mesoderm lineage differentiation. (a) Schematic diagram of experimental strategy was illustrated here. (b) Size and morphologies of EBs in unstimulated EBs (Dox -) and STAT3 KO EBs (Dox +). Following STAT3 deletion, the size of STAT3 KO EBs (Dox +) was not significantly different compared to unstimulated EBs (Dox -), suggesting that STAT3 has no effect on the cell growth and morphology of EBs formed when STAT3 was deleted. (c) Deletion of STAT3 led to significant reduction in the expression of mesodermal markers when STAT3 was deleted from day 0 onwards of cardiomyocyte differentiation of ESCs. Number of EB clones used for qPCR was 30 EBs at each time point.

ESCs were dissociated using 0.1% trypsin-EDTA (Gibco, Grand Island, New York, USA) and suspended in Glasgow Minimum Essential Medium supplemented with 15% Tet system approved fetal bovine serum (A Takara Bio Company, Mountain View, CA, USA), 1 mM sodium pyruvate, 0.1 mM nonessential amino acids, 0.1 mM 2-mercaptoethanol, 50 U/ml penicillin, 50 ug/ml streptomycin, and 50 ug/ml ascorbic acid (Sigma-Aldrich, Saint Louis, Missouri, USA) (EB medium) [43]. ESCs were differentiated to form embryoid bodies (EBs) using the hanging drop method as previously reported [44]. Hanging drops each of 25 ul, containing 1000 embryonic stem cells were seeded on the lid of 10 cm^2^ cell culture dish, and the dish was filled with PBS to prevent desiccation of EBs. Hanging drops were incubated at 37°C in humidified air with 5% CO_2_ for 3 days. EBs were then seeded on gelatin-coated plate on the 4th day of differentiation. Dox was added to differentiation medium to a final concentration of 1 ug/ml at the corresponding day indicated in the diagram (Figure 1(a)). EB medium was changed every two days. The beating cardiomyocytes could be observed as early as day 8.

### 2.9. Microscopy and Imaging

Images of inducible ESCs were captured with a Zeiss AxioVision 4.7 or Nikon A1R-A1 confocal microscopy system. Images were taken after 24 hours Dox induction.

## 3. Results

### 3.1. Generation of Inducible STAT3 KO and STAT3 CA ESCs

**Figure 2:**
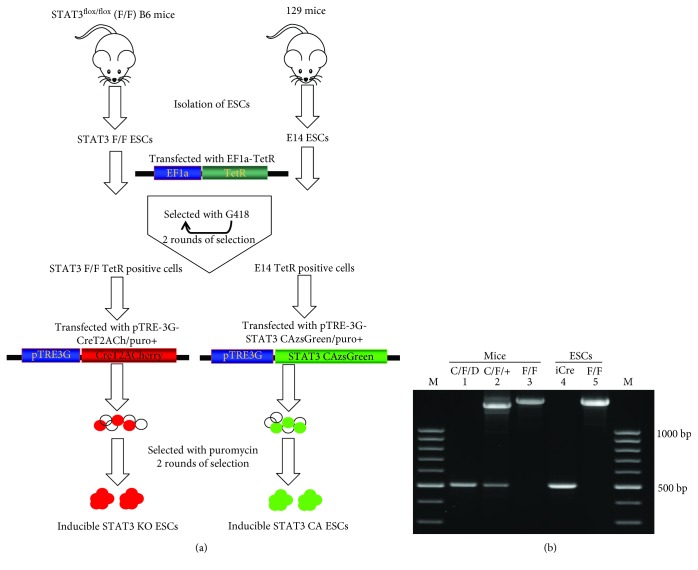
Generation of inducible STAT3 KO and STAT3 CA ESCs. (a) Schematic chart for selection of inducible STAT3 KO and STAT3 CA ESCs. (b) Ablation of STAT3 genomic DNA in inducible STAT3 KO ESCs was detected as a 480 base pair fragment. The hearts from the cardiac-specific STAT3 KO mice (C/F/D) (lane 1, both STAT3 alleles were deleted), heterozygous (C/F/+) (lane 2, one wild-type STAT3 allele and one deleted STAT3 allele), and STAT3^flox/flox^ mice (F/F) (lane 3, both STAT3 alleles with two *lox*P sites inserted) were used. Inducible Cre (iCre) (lane 4, STAT3 was deleted after Dox induction) and STAT3^flox/flox^ (F/F) ESCs (lane 5, both STAT3 alleles with two *lox*P sites inserted) were used.

To determine the biological functions of STAT3 in ESCs, we generated the transgenic mouse ESCs harboring a Dox-inducible Cre mCherry transgene and STAT3 CA GFP transgene using an inducible Tet-on system, designed as InSTAT3 KO and InSTAT3 CA ESCs. The regulatory vector pEF1a-Tet was transfected into the mouse STAT3^flox/flox^ (F/F) [45] ESCs or wild-type ESCs E14. Following 2 rounds of selection in G418, these G418-resistant cell clones were transfected with pTRE3G-CreT2ACherry, and pTRE3G-STAT3 CA-IRESzsGreen, respectively (Figure S1). Selected by puromycin, the transgenic cell clones which bear Dox-inducible Cre and STAT3 CA Tet-on positive ESC clones were obtained and propagated. In these clones, mCherry or GFP, indicating STAT3-KO or pSTAT3-overexpression, could be turned on or off by Dox adding or removing (Figure 2(a)).

STAT3 deleted (STAT3^D^) alleles were analysed by PCR according to the previous study [45] using primer pairs 1 and 3 (Table S3). The STAT3^D^ was detected as a 480 base pair fragment. Inducible Cre (iCre) ESCs following 24 hours Dox induction and STAT3^flox/flox^ (F/F) ESCs were genotyped. Mouse heart tissue samples which included the hearts from STAT3-deficient mice (C/F/D), heterozygous (C/F/+), and STAT3^flox/flox^ mice (F/F) were used as positive control. The STAT3^D^ allele was detected as a 480 bp fragment in STAT3-deficient heart (C/F/D) and STAT3 KO ESCs (Figure 2(b) lanes 1 and 4). With this primer pair, it amplified an approximately 1.5 kb fragment and a 480 bp in heterozygous (C/F/+) heart sample (Figure 2(b) lane 2), whereas only a 1.5 kb fragment was detected in the STAT3^flox/flox^ (F/F) heart and F/F ESCs (Figure 2(b) lanes 3 and 5).

### 3.2. Recombinant ESCs Allowing Dox-Inducible Deletion of STAT3 (InSTAT3 KO) or pSTAT3 Overexpression (InSTAT3 CA)

**Figure 3:**
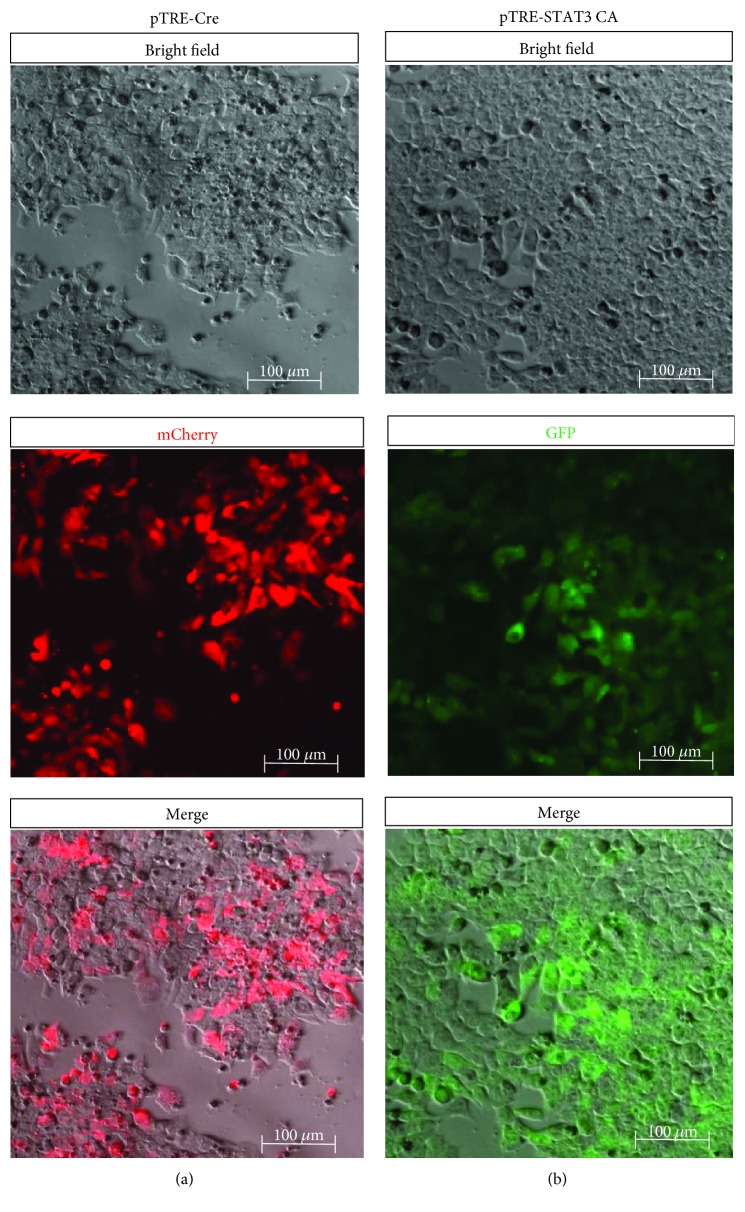
Recombinant ESCs allowing Dox-inducible deletion of STAT3 (InSTAT3 KO) or pSTAT3 overexpression (InSTAT3 CA). (a) In pTRE-CreCh/pEF1aTetR-inducible STAT3 KO ESCs, mCherry was observed after 24 hours Dox induction, indicating that Cre was expressed and functioned to delete STAT3 *in vitro*. (b) In pTRE-STAT3 CA/pEF1aTetR-inducible STAT3 CA ESCs, GFP was expressed after 24 hours Dox induction, suggesting that STAT3 CA was expressed.

In this InSTAT3 KO and InSTAT3 CA ESC system, STAT3 can be temporally and specifically induced or deleted upon Dox addition. Inducible Cre and STAT3 CA Tet-on positive ESC clones were selected and propagated. Following 24 hours Dox stimulation, strong expression of Cre-T2A-mCherry (Figure 3(a)) and STAT3 CA-IRES-GFP (Figure 3(b)) was observed. Detection of mCherry expression represented that Cre gene was induced, and its recombinase activity was functional to delete STAT3^flox/flox^ *in vitro*, resulting in a mutant STAT3 protein missing the Src-homology2 (SH2) domain which is essential for STAT3 function (Figure 3(a)), whereas GFP expression indicated that STAT3 was constitutively activated in ESCs (Figure 3(b)).

### 3.3. pSTAT3 and STAT3 Protein Expressions Can Effectively Respond to Dox Treatment

**Figure 4:**
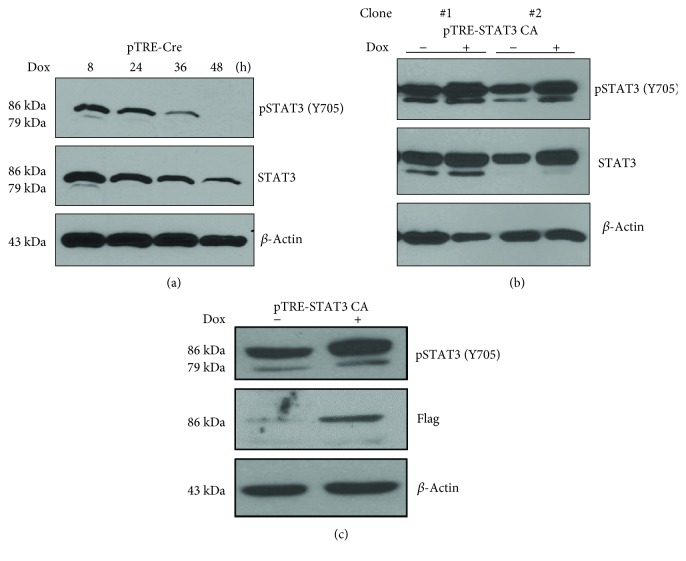
pSTAT3 and STAT3 protein expressions can effectively respond to Dox induction. (a) In pTRE-CreCh/pEF1aTetR ESCs, after the Dox induction, pSTAT3 was reduced and undetected after 48 hours induction, while STAT3 was decreased but still detectable after 48 hours. (b) In pTRE-STAT3 CA/pEF1aTetR ESCs, pSTAT3 and STAT3 were rapidly upregulated after 24 hours Dox induction. (c) In pTRE-STAT3 CA/pEF1aTetR ESCs, after the Dox induction, flagged-STAT3 CA expression was detected, which indicated that CA-STAT3 was expressed in these cells.

Through Western blot analysis, the expression levels of pSTAT3 and STAT3 were examined in these inducible ESCs after Dox induction. The results showed that in pTRE-Cre ESCs, pSTAT3 protein level was efficiently ablated and undetected while STAT3 protein level was decreased but still detectable after 48 hours Dox induction (Figure 4(a)). In pTRE-STAT3 CA ESCs, pSTAT3 was rapidly upregulated after 24 hours Dox induction (Figure 4(b)). Since STAT3 antibody can detect total STAT3 which included both pSTAT3 and STAT3 proteins, therefore, STAT3 protein level was upregulated slightly upon Dox induction due to the increased expression of pSTAT3 protein level. Moreover, the observations showed that clone #1 had slightly higher level of pSTAT3 induction as compared to clone #2 upon Dox induction (Figure 4(b)). Flagged-STAT3 CA expression was detected using anti-flag antibody. STAT3 CA was rapidly upregulated after Dox stimulation but undetectable in control (Figure 4(c)).

### 3.4. Use of InSTAT3 KO ESCs to Study Mesoderm Lineage Differentiation

Next, inducible STAT3 KO system was utilized to identify the role of STAT3 in ESC lineage differentiation. The effect of deletion of STAT3 on cardiomyocyte differentiation of ESCs was examined. Schematic diagram of experimental strategy was illustrated here (Figure 1(a)). STAT3 was deleted from day 0 onwards of cardiomyocyte differentiation of ESCs. Undifferentiated ESCs and differentiated EBs were collected at day 0, 3, 6, 9, 12, and day 15, and qPCR was performed to analyze the expression of mesodermal markers and cardiac transcription factors in these STAT3 KO-differentiated EBs as compared to uninduced EBs. Also, morphology of EBs formed when STAT3 was deleted from D0 was monitored.

In order to examine whether the deletion of STAT3 had any effect on the formation of EBs, the morphology of EBs formed when STAT3 was deleted from day 0 onwards of cardiomyocyte differentiation from ESCs was first observed. Pictures of Cre-inducible EBs 48 hours following Dox induction showed not much difference in the size of STAT3 KO EBs as compared to unstimulated EBs (Figure 1(b)), suggesting that STAT3 has no effect on the cell growth and morphology of EBs formed when STAT3 was deleted.

Time course qRT-PCR suggested that deletion of STAT3 resulted in decreased expression of mesodermal markers such as T-Brachyury, Mesp1, Fgf5, and Hand1 during differentiation as compared to uninduced EBs (Figure 1(c)). Together, these results demonstrated that STAT3 is required for early ESC differentiation into cardiac-committed mesoderm.

### 3.5. STAT3 Deletion May Affect Cardiomyocyte Differentiation

**Figure 5:**
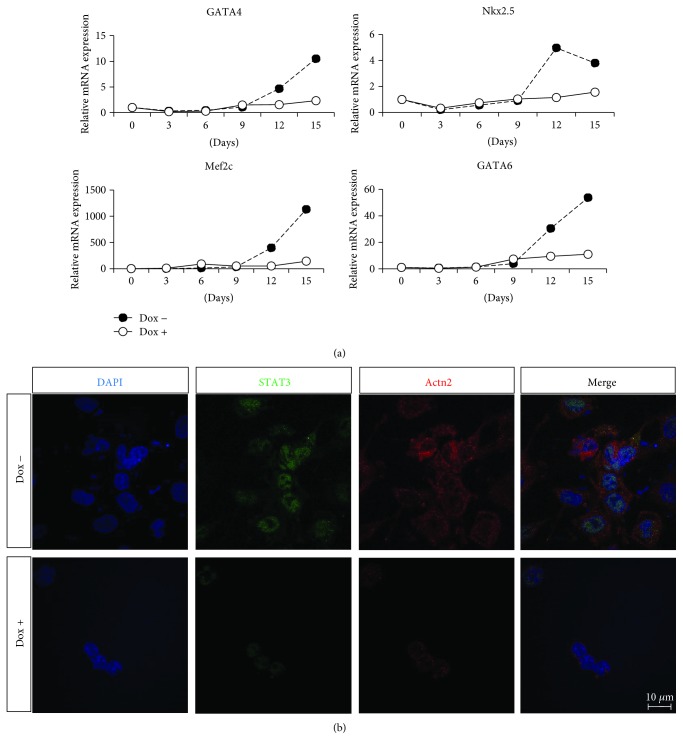
STAT3 deletion may affect cardiomyocyte differentiation. (a) Deletion of STAT3 led to significant reduction in the expression of cardiac transcription factors when STAT3 was deleted from day 0 onwards of cardiomyocyte differentiation of ESCs, indicating that STAT3 is required for cardiomyocyte differentiation of ESCs. (b) Decreased expression of STAT3 and Actn2 following STAT3 deletion on D0. Number of EB clones used for qPCR was 30 EBs at each time point.

Time course qRT-PCR revealed that deletion of STAT3 led to significant reduction in the expression of cardiac transcription factors such as GATA4, Mef2c, Nkx2.5, and GATA6 (Figure 5(a)). Next, D13 EBs were costained with STAT3 and cardiac specific marker Actn2 to examine the expression of STAT3 when Dox was added on D0 of cardiomyocyte differentiation of ESCs. Immunofluorescent staining of STAT3 KO EBs demonstrated reduced expression of STAT3 and cardiac specific marker, Actn2, as compared to uninduced EBs (Figure 5(b)). Together, these results demonstrated that STAT3 is required for ESC differentiation into cardiac lineage.

## 4. Discussion

Studies have demonstrated that STAT3 signaling is essential in ESC pluripotency maintenance, embryogenesis, and cell-fate determination in mammals. However, it is difficult to analyze STAT3 functions in the initiation of cardiomyogenesis because STAT3 knockout resulted in early embryonically lethal at the time of cardiomyocyte formation at E7.5–E8.5 [1, 20]. Likewise, it was difficult to elucidate early molecular events in mammalian embryogenesis using *in vivo* mammalian systems. Therefore, ESC is an ideal alternative model to investigate the role and/or targets of STAT3 during embryogenesis through differentiation of ESCs into defined cell types.

In the present study, we have established an *in vitro* inducible STAT3 knockout and pSTAT3 overexpressed ESC system in which STAT3 expression can be strictly controlled by Dox stimulation, which facilitate the study of the direct functions or targets of STAT3 during modulation of ESC differentiation (Figure 2(a)). Genotyping of inducible STAT3 KO ESCs following 24 hours Dox stimulation showed deletion of STAT3 alleles (Figure 2(b)), similar to the previous study [45]. It was further confirmed by Western blot that pSTAT3 and STAT3 protein levels were significantly reduced in InSTAT3 KO ESCs while tremendously upregulated in InSTAT3 CA ESCs following Dox stimulation (Figure 4). Thus far, our study provides the first comprehensive system to study STAT3 function in ESC pluripotency maintenance using the well-established *in vitro* inducible STAT3 knockout and pSTAT3 overexpressed ESC system.

In addition, we have carried out microarray experiments to analyze the transcriptomic profiles in Dox-inducible STAT3 CA and STAT3 KO mESCs after Dox induction (unpublished data). Some of the regulated genes have been validated by qRT-PCR, such as suppression of cytokine signaling 3 (SOCS3), inhibitor of DNA-binding 1 (Id1), and GATA-binding protein 6 (GATA6). SOCS3 expression was upregulated upon overexpression of pSTAT3 while downregulated when STAT3 was KO (Figure S2). Id1 is a direct target gene transcriptionally induced by STAT3 [46]. In our study, Id1 expression was upregulated upon overexpression of pSTAT3 while downregulated when STAT3 was KO. Likewise, GATA-4, -5, and -6 are expressed in cardiac tissues [47] and endoderm-derived tissues [48–51]. Studies using knockout mice have proved that GATA4 and GATA6 are crucial for early development of the heart. Inactivation of GATA4 in transgenic mice causes embryo lethal between day 8 and 9 post coitum due to the failure of heart tube formation, implying that GATA4 is crucial for normal heart development [52, 53]. GATA6 deficient mice embryos would die shortly before the heart induction at E5.5–E7.5 [54, 55]. Therefore, expression of GATA4 and GATA6 in the present study could be used to trace the development of cardiac differentiation (Figure 5(a)).

In vertebrates, distinct sets of cardiac transcription factors to control gene expression in the developing heart are represented by the GATA family, the homeobox Nkx2.5 transcription factors, and the myocyte enhancer factor 2 (Mef2) transcription factors. Nkx2.5 and Mef2c individual mutants display early embryonic lethality and defeats in cardiac looping morphogenesis [56–59]. Moreover, several studies have shown that Nkx2.5 and Mef2c act in combination to regulate heart development [60, 61]. Additionally, the presence of essential GATA binding sites has been reported in the enhancers of Nkx2.5 and Mef2c [62–64]. Therefore, it exists a positive regulatory loop between GATA, Nkx2.5, and Mef2c that regulates cardiomyogenesis.

Several studies have demonstrated that mesoderm posterior basic helix-loop-helix transcription factor 1 (Mesp1) significantly promotes cardiovascular differentiation during embryonic development and pluripotent stem cell differentiation. Importantly, it has been proved that Mesp1 resides at the top of the cellular and transcriptional hierarchy that orchestrates multipotent cardiovascular progenitor (MCP) specification [65, 66]. Thus, Mesp1 is a specific marker for cardiac mesoderm, and it was shown that Mesp1 could be modulated by STAT3 expression (Figure 1(c)). Also, functions of the basic helix-loop-helix transcription factors (Hand1) and the T-box transcription factor Brachyury (T-Brachyury) have been implicated in cardiac morphogenesis and cardiomyocyte differentiation [67–71].

Importantly, the inducible ESCs' capability of cardiac differentiation has been briefly examined. The results showed that InSTAT3 KO mESCs could be differentiated into beating cardiomyocytes at 9th day of EB culture. While STAT3 was deleted at day 0 (Dox was added at day 0), no beating cardiomyocytes was observed. According to these observations, it can thus be concluded that STAT3 is involved in initial stages of cardiac differentiation of ESCs. In conclusion, inducible STAT3 CA and STAT3 KO ESC system established in the present study could provide an excellent tool to demonstrate the functions or targets of STAT3 during modulation of ESC differentiation into certain tissue progenitor cells. Moreover, it has been reported that inhibition of STAT3 activity leads to downregulation of key specific cardiac genes [26]. Likewise, another study demonstrated that specific dose of LIF and BMP2 could efficiently differentiate mESCs into cardiomyocytes via their synergistic activation of STAT3 signal transduction pathway [72]. Besides, it has been proved that STAT3 plays a key role in ESC self-renewal and proliferation through interacting with other stem cell factors, such as Oct4 and Nanog, especially, STAT3 is found to bind to both ESCs enriched genes and developmentally repressed genes in ESCs [73, 74]. Although the mechanisms of STAT3 signaling pathways in ESC regulation have been intensively studied [75–79], the direct targets of STAT3 signaling pathways have not yet been fully elaborated. The controllable system of STAT3 expressed in this study provides the opportunity to find out the direct genes and pathways modulated by STAT3 in maintaining ESC pluripotency and cell-fate determination.

Furthermore, through partial gain- or loss-of-function approaches, the transcription profiles of STAT3 expression in ESCs were reported. Mouse STAT3 overexpressing ESCs which composed of the entire coding regions of mouse STAT3 to increase overall STAT3 expression was profiled [80, 81]. Therefore, phosphorylation of STAT3 is required for translocation of STAT3 into nucleus to regulate gene expression. Whereas the InSTAT3 CA ESCs in this study could constitutively express the phosphorylated STAT3 in the nucleus, making STAT3 directly function in the regulatory networks of ESCs. Similarly, another analysis of transcriptomic profiles were carried out in STAT3 dominant-negative mutant ESCs where only a 50% reduction of endogenous STAT3 activation happened with incomplete silencing of STAT3 expression [26, 82, 83]. Also, in some studies, JAK1 and JAK2 inhibitors were used to identify the mechanisms of STAT3 signaling pathways indirectly and its downstream targets that regulate self-renewal in ESCs [84, 85]. Similarly, JAK2 inhibitor or STAT3 inhibitor were used to demonstrate that STAT3 is crucial in ESC differentiation [72, 86]. Likewise, RNAi technique was utilized to examine if STAT3 is essential for neuronal differentiation [87]. Nonetheless, the main drawback associated with these strategies is the specificity of the inhibitors and siRNA and thus suffers from off-target effects [88, 89]. In addition, STAT3 activity is not completely abolished using these approaches. Alternatively, in the present study, it was indicated that STAT3 was completely deleted in InSTAT3 KO cells, and hence, the factors affected were solely due to the deletion of STAT3, not other unknown off-targets. Taken together, the inducible STAT3 ESCs in the present study would provide a unique tool for investigation of direct targets and pathways, whereby STAT3 regulates stem cell development and differentiation into somatic progenitors.

## 5. Conclusion

In summary, the present study has provided an *in vitro* inducible STAT3 knockout and pSTAT3 overexpressed ESC system where STAT3 expression level can be strictly controlled by Dox stimulation. This allows the study of STAT3 functions in ESC pluripotency maintenance. Moreover, it has provided another unique tool to demonstrate STAT3 functions involved in ESC differentiation into various lineages of cells. Taken together, the results of this study provide the opportunity and basis for further elaboration of the mechanisms, whereby STAT3 maintains ESC pluripotency and regulates ESC differentiation during mammalian organogenesis.

## Data Availability

The data used to support the findings of this study are available from the corresponding author upon request.

## Abbreviations

CA-STAT3: Constitutive activated STAT3
cDNA: Complementary DNA
Dox: Doxycycline
ESCs: Embryonic stem cells
EB: Embryoid body
GFP: Green fluorescent protein
GOI: Gene of interest
KO: Knockout
LIF: Leukemia inhibitory factor
Nanog: Nanog homeobox
OCT4: Octamer-binding transcription factor 4
PCR: Polymerase chain reaction
qRT-PCR: Quantitative reverse transcriptase-PCR
SH2: Src-homology2
SOCS: Suppressor of cytokine signaling
SOX2: SRY-related HMG box
STAT: Signal transducer and activator of transcription
STAT3 pY705: Tyrosine 705 phosphorylation
TetR: Tetracycline-control transcriptional activation.

## Supplementary Materials

Supplementary Materials Figure S1: pTRE3G-IRES Tet-on System and major constructs of pTRE3G-IRES Tet-on System. Figure S2: validation of SOCS3 gene in InSTAT3 KO and CA mESCs by qRT-PCR. Table S1: list of primers used in genotyping. Table S2: list of primers used in qRT-PCR. Table S3: list of antibodies used in this study.
