# Supplementary material for: STAT3-Inducible Mouse ESCs: A Model to Study the Role of STAT3 in ESC Maintenance and Lineage Differentiation
Source: Stem Cells Int. 2018 Sep 4;2018:8632950. doi: 10.1155/2018/8632950 (PMC6142778; doi:10.1155/2018/8632950)
Supplement: Supplementary Materials — Figure S1: pTRE3G-IRES Tet-on System and major constructs of pTRE3G-IRES Tet-on System. Figure S2: validation of SOCS3 gene in InSTAT3 KO and CA mESCs by qRT-PCR. Table S1: list of primers used in genotyping. Table S2: list of primers used in qRT-PCR. Table S3: list of antibodies used in this study. [file 8632950.f1.docx]

# Supplemental Figures

**Preparation of pTRE3G-Cre and pTRE3G-STAT3 CA plasmids**

NlsCre was purchased from commercial entities and Constitutive activated STAT3 (STAT3 CA) was obtained from pJX40-CA kindly provided by Professor Cao Xinmin [42]), were cloned into a TetR response vector controlled by the tetracycline responsive element (pTRE-), fused with C-T2ACherry and C-IRESzsGreen respectively.

**
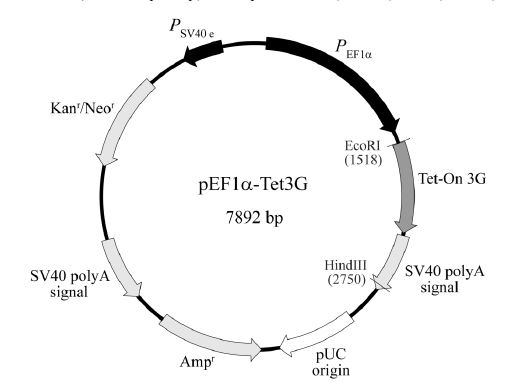
**

pTRE3G-NlsCreT2A-Cherry

5212 bp

NlsCreT2A-

Cherry

**Amp(R)**

**SV40 polyA signal**

**pTRE3G**

**pUC origin**

*Eco* RI (5208)

*Apa* LI (3636)

*Apa* LI (4882)

*Ava* I (2)

A

B

C

pTRE3G-mSTAT3 CA IRES-zsGreen

5683 bp

mSTAT3 CA IRES-zsGreen

**Amp(R)**

**SV40 polyA signal**

**pTRE3G**

**pUC origin**

*Bam* HI (2700)

*Eco* RI (5679)

*Sal* I (384)

*Apa* LI (4107)

*Apa* LI (5353)

*Ava* I (2)

**Figure S1. pTRE3G-IRES Tet-on System and major constructs of pTRE3G-IRES Tet-on System.**

The 3^rd^ generation Tet-on System was purchased from Clontech Laboratories (Cat. no. 631167). This Tet-On 3G System is inducible gene expression system for mammalian cells. The system consists of the Tet-On 3G transactivator, TetR (Figure S1A) and contains a gene of interest (GOI) under the control of a TRE3G promoter (pTRE3G) (Figure S1B, 1C) which will express high levels of GOI driven by TetR with doxycycline induction.

**Validation of SOCS3 gene in InSTAT3 KO and CA mESCs by qRT-PCR**

We have carried out microarray experiments to analyze the transcriptomic profiles in Dox-inducible STAT3 CA and STAT3 KO mESCs after Dox induction (unpublished data). SOCS3 expression was upregulated upon overexpression of pSTAT3 (Microarray: 2.24 fold) while downregulated when STAT3 was KO (Microarray: 0.23 fold). Some regulated genes such as SOCS (shown as below) have been validated by qPCR and results of qPCR confirms microarray data whereby SOCS3 expression was upregulated upon overexpression of pSTAT3 (qPCR Figure S2A: 2.55 fold) while downregulated when STAT3 was KO (qPCR Figure S2B: 0.21 fold).

**A B**

**Figure S2. Validation of SOCS3 gene in In STAT3 KO and CA mESCs by qRT-PCR.**

SOCS3 expression has been validated by qPCR and results of qPCR confirms microarray data. SOCS3 expression was upregulated upon overexpression of pSTAT3 (Figure S2A) while downregulated when STAT3 was KO (Figure S2B).

**Supplemental Tables**

**Table S1.** List of primers used in genotyping

| Gene (Mouse) | primer |
| --- | --- |
| Primer 1 | 5′-ATT GGA ACC TGG GAC CAA GTG G |
| Primer 3 | 5′-GCT GGC TCA TAG GCA AAA ACA C |

**Table S2.** List of primers used in qRT-PCR

| Gene (Mouse) | Forward primer (5'- 3') | Reverse primer (5'- 3') |
| --- | --- | --- |
| T-Bra | CTCCAACCTATGCGGACAAT | CCCCTTCATACATCGGAGAA |
| Mesp1 | GTCTGCAGCGGGGTGTCGTG | CGGCGGCGTCCAGGTTTCTA |
| Fgf5 | TGCTGTGTCTCAGGGGATTG | TCCGTAAATTTGGCACTTGCAT |
| Hand1 | TCGCCTACTTGATGGACGTG | CTGCTGAGGCAACTCCCTTTTC |
| GATA4 | CCACGGGCCCTCCATCCAT | GGCCCCCACGTCCCAAGTC |
| Mef2c | GCTGAGATACGCTTAGCACTTTGAGT | CGGGTCTGTCCAAACCTCTATACA |
| Nkx2.5 | CTCCGATCCATCCCACTTTA | AGTGTGGAATCCGTCGAAAG |
| GATA6 | TGTGCAATGCATGCGGTCTCTACAGCA | TTCATAGCAAGTGGTCGAGGCACCC |
| SOCS | ATGGTCACCCACAGCAAGTTT | TCCAGTAGAATCCGCTCTCCT |

**Table S3.** List of antibodies used in this study

| Antibody name | Protein | Antibody type | Supplier | Catalogue No. | Application & Dilution |
| --- | --- | --- | --- | --- | --- |
| pSTAT3 (Tyr705) | Phospho-Signal transducer and activator of transcription 3 | Rabbit polyclonal | Cell Signaling | #9131 | WB: 1:1000 |
| STAT3 | Signal transducer and activator of transcription 3 | Mouse monoclonal | Cell Signaling | #9139 | WB: 1:1000  IF: 1:500 |
| Flag | Flag-taq | Mouse monoclonal | Sigma Aldrich | F3165 | WB: 1:1000 |
| Actn2 | Actinin alpha 2 | Mouse monoclonal | Sigma Aldrich | A7811 | IF: 1:500 |
| β-Actin | Actin beta | Mouse monoclonal | Santa Cruz | Sc-47778 | WB: 1:5000 |
